# Chronic Diabetic Wounds and Their Treatment with Skin Substitutes

**DOI:** 10.3390/cells10030655

**Published:** 2021-03-15

**Authors:** Jordan Holl, Cezary Kowalewski, Zbigniew Zimek, Piotr Fiedor, Artur Kaminski, Tomasz Oldak, Marcin Moniuszko, Andrzej Eljaszewicz

**Affiliations:** 1 Department of Regenerative Medicine and Immune Regulation, Medical University of Bialystok, 15-269 Bialystok, Poland; Jordanm.Holl@umb.edu.pl; 2 Department of Dermatology and Immunodermatology, Medical University of Warsaw, 02-008 Warsaw, Poland; ckowalewski@wum.edu.pl; 3 Institute of Nuclear Chemistry and Technology, 03-195 Warsaw, Poland; z.zimek@ichtj.waw.pl; 4 Department of General and Transplantation Surgery, Medical University of Warsaw, 02-006 Warsaw, Poland; piotrfiedor@wp.pl; 5 Department of Transplantology and Central Tissue Bank, Medical University of Warsaw, 02-091 Warsaw, Poland; Artur.Kaminski@wum.edu.pl; 6 Polish Stem Cell Bank (PBKM), 00-867 Warsaw, Poland; Tomasz.Oldak@pbkm.pl; 7 Department of Allergology and Internal Medicine, Medical University of Białystok, 15-276 Białystok, Poland

**Keywords:** wound healing, diabetes, chronic wounds, skin substitutes, skin dressings, matrices

## Abstract

With the global prevalence of type 2 diabetes mellitus steeply rising, instances of chronic, hard-healing, or non-healing diabetic wounds and ulcers are predicted to increase. The growing understanding of healing and regenerative mechanisms has elucidated critical regulators of this process, including key cellular and humoral components. Despite this, the management and successful treatment of diabetic wounds represents a significant therapeutic challenge. To this end, the development of novel therapies and biological dressings has gained increased interest. Here we review key differences between normal and chronic non-healing diabetic wounds, and elaborate on recent advances in wound healing treatments with a particular focus on biological dressings and their effect on key wound healing pathways.

## 1. Introduction

The global impact of diabetes, including type 2 diabetes mellitus (T2DM), is severe, costing over 760 billion dollars—constituting 10% of adults’ annual health expenditure. More importantly, diabetes is projected to affect over 700 million individuals by 2045 (7.8% of the global population) [1,2]. In 2019 alone, more than 4 million adults died from direct and associated complications of diabetes. This prevalence and burden clearly outline diabetes and its associated complications as pressing global concerns.

Diabetic patients develop wounds characterized by impaired healing, prolonged inflammation, and reduced epithelization kinetics. Notably, 15% of patients suffering from T2DM develop ulcers localized on the lower limbs, referred to as diabetic foot ulcers (DFUs). DFUs represent the most severe form of diabetic wounds which may lead to lower limb amputation or death [3]. In fact, DFUs precede 84% of all diabetes-related lower limb amputations. Therefore, there exists a substantial need to elucidate the pathological processes causing ulceration, and which affect wound healing in diabetics.

Wound healing is defined as a natural physiological process occurring as the reaction to structural damage of tissues, including skin. These mechanisms involve sophisticated complimentary interactions between different cell types, acting through networks of soluble mediators, including cytokines, chemokines, growth factors, and metabolites. Wound healing consists of four subsequent and overlapping phases: hemostasis, inflammation, proliferation (re-epithelization), and remodeling (scar maturation).

Interestingly, diabetic hyperglycemia contributes to a variety of systemic complications, causing an array of local pathologies manifesting within the wound microenvironment, including chronic inflammation, dysregulated angiogenesis, hypoxia-induced oxidative stress, neuropathy, advanced glycation end-products, and impaired neuropeptide signaling [4]. Here we discuss the influence of diabetes on wound healing and the formation of diabetic foot ulcers. Moreover, we discuss strategies for diabetic wound treatment concentrated on the use of skin substitutes and biological dressings.

## 2. Wound Healing Starts with Homeostasis

Immediately after wounding, degranulation of mast cells induces capillary permeability, in addition to vasodilation, increasing bleeding and allowing the influx of immune cells. Furthermore, the coagulation system is activated, and a scab is formed of provisional components [5]. Simultaneously, activated keratinocytes, fibroblasts, and platelets release soluble mediators: (a) growth factors, such as platelet-derived growth factor (PDGF), epidermal growth factor (EGF), and vascular endothelial growth factor (VEGF); (b) chemokines, including IL-8 (CXCL-8) and CXCL-2; (c) danger-associated molecular patterns (DAMPs) such as histones, genomic DNA, adenosine 5′-triphosphate (ATP), high mobility group box protein 1 (HMGB1); and (d) cytokines; namely, thymic stromal lymphopoietin (TSLP), IL-33, and IL-25 [6,7,8,9]. Notably, all of the above-mentioned inflammatory mediators act as danger signals. Consequently, they trigger the infiltration of patrolling inflammatory cells and the induction of local immune responses (inflammation phase) and subsequent proliferative induction of tissue-resident cells.

## 3. Wound Inflammation Orchestrates Healing and Regeneration

**Figure 1:**
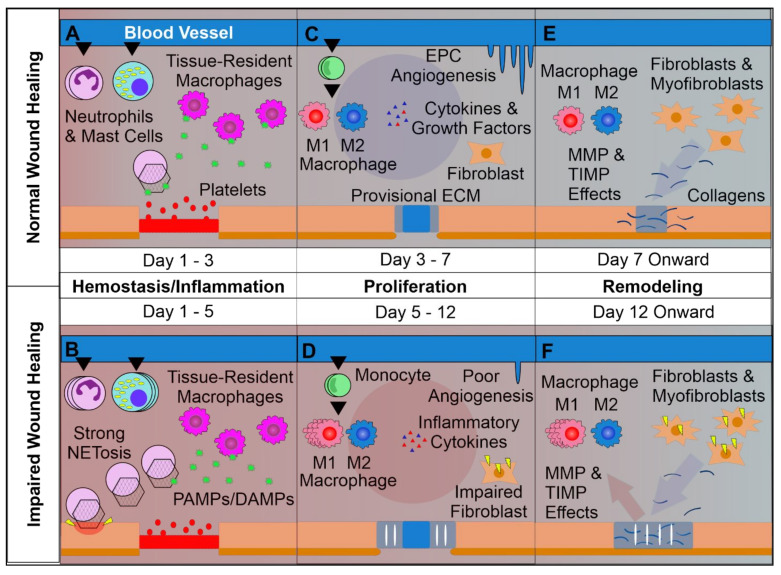
Overview of Normal vs. Impaired Wound Healing. (**A**): The first phase of wound healing is hemostasis. Platelets form a clot at the site of injury, and chemoattractants are released, recruiting key inflammatory cells. Next, inflammation takes charge, with infiltrating neutrophils and mast cells releasing pro-inflammatory cytokines and inducing strong sanitizing effects. This is accompanied by neutrophil extracellular trap (NETosis) induction, which assists in capturing and destroying invading pathogens. Tissue-resident macrophages react to pathogen- and damage-associated molecular patterns (PAMPs & DAMPs), activating. Later a provisional matrix comprised of fibronectin and other provisional extracellular matrix (ECM) components forms from the clot. (**B**): Impaired wounds see an upregulated influx of neutrophils and mast cells, leading to an overactive inflammatory response, causing collateral damage and extending the inflammatory phase to the detriment of subsequent phases. (**C**): Following resolution of strong inflammation, the proliferative phase begins. Crucially, endothelial progenitor cells are stimulated by growth factors to induce angiogenesis. This angiogenesis allows for wound-resident cells to be supplied with oxygen and nutrients, facilitating their function. Infiltrating monocytes differentiate into M1 and M2 macrophage subsets. M1 macrophages maintain a strong inflammatory profile, but are counterbalanced by pro-regenerative M2 macrophages which release anti-inflammatory cytokines, growth factors, and proteases which replace the provisional ECM with collagens, assisted by properly functioning fibroblasts. This process results in thick granular tissue and full keratinocyte coverage. (**D**): Impaired wounds result in poor angiogenesis and, in the case of T2DM, glycated proteins. This hypoxic environment induces oxidative stress, driving inflammatory M1 macrophage polarization and impairment of fibroblasts, resulting in poor ECM reorganization and a persistent inflammatory environment. (**E**): Remodeling is carried out by macrophages, fibroblasts, and myofibroblasts re-organizing the provisional ECM into a coherent scar structure primarily by means of matrix metalloproteinases (MMPs) and their inhibitors (TIMPs), resulting in tissue with strong tensile strength and functionality. (**F**): Impaired wound-resident cells remain ineffective and pro-inflammatory. Collagen reorganization resolves poorly, resulting in weak, non-functional skin that is apt to re-injure and potentially ulcerate, perpetually inflamed.

A strong inflammatory cascade, initiated during hemostasis, now commences to clean the wound of debris, damaged cells, and microbes. The inflammatory phase is characterized by (a) an influx of inflammatory cells including neutrophils, monocytes/macrophages, mast cells, and T cells; (b) the accumulation of inflammatory mediators such as cytokines, chemokines, and lipid mediators; and c) the release of extracellular matrix degradation enzymes such as matrix metalloproteases (MMPs) and collagenases; causing swelling, heat, and pain (Figure 1) [4].

It is widely recognized that localized, properly controlled inflammation acts as a trigger for the proliferative and remodeling phases [10,11]. On the other hand, the uncontrolled or prolonged inflammatory responses frequently observed in diabetic wounds lead to the impairment of subsequent phases of the wound healing process, or are implicated as a contributor to ulceration [12,13]. Local inflammation is strictly associated with neutrophil infiltration and activation. Interestingly, neutrophils are absent in unwounded skin and their trafficking to the wound area is induced and controlled by tissue-resident T cells, mast cells, and macrophages [14]. In fact, neutrophils represent an important source of proteases (including elastase, cathepsin G, and urokinase-type plasminogen activator—that support re-epithelialization) [15,16,17]; reactive oxygen and nitrogen species, cytokines (including IL-1β, tumor necrosis factor (TNF), IL-6, IL-12p40, and transforming growth factor β (TGF-β)), and chemokines (including CCL2, CCL3, CCL5, CXCL1, and CXCL2) [18]. Moreover, generally high neutrophil counts within the wound, and the consequently increased neutrophil-to-lymphocyte ratio is recognized as a hallmark of impaired wound healing observed in T2DM-affected individuals [19]. Interestingly, T2DM is known to induce neutrophil extracellular trap induction (NETosis), a phenomenon which may be responsible for delayed wound healing, given that disruption of neutrophil ability to undergo NETosis led to accelerated wound closure in previous studies (Figure 1) [12,20]. This continual activation of neutrophils and induction of NETosis results in the induction of yet more inflammation by way of mitochondrial DNA and histone H4 [21] in contrast with the normal process of inflammatory resolution by way of neutrophilic apoptotic body phagocytosis [11]. This uptake of neutrophil-derived apoptotic bodies’ by infiltrating monocytes/macrophages helps resolve the inflammatory phase in a self-perpetuating manner by limiting inflammatory cell infiltration and shifting the production of eicosanoids from pro-inflammatory to anti-inflammatory mediators [22,23,24]. Unfortunately, however, in diabetic wounds the inflammatory phase is significantly prolonged by the disruption of mechanisms which both control the influx of neutrophils as well as regulate their inflammatory processes [12,21,25]. Interestingly, it seems that the cause of many observed dysregulations of the inflammatory phase is not directly associated with localized high glucose levels but rather the epigenetic polarization of innate immune cell pro-inflammatory function prior to wound infiltration, as in progenitor cell modification due to T2DM-related systemic complications such as hyperglycemia [26]. This polarization of innate immune cells towards pro-inflammatory phenotypes is additionally supported by systemic inflammatory effects observed in diabetic patients and animal models [27]. However, to date, mechanisms controlling the epigenetic regulation of neutrophils and monocytes/macrophages in diabetic individuals remain elusive.

Monocyte migration to injured skin is controlled by chemokines derived by mast cells, keratinocytes, and fibroblasts acting through CCR2, CCR5, and mainly monocyte chemotactic protein 1 (MCP-1) [28]. Notably, due to pleiotropic biological activities, monocytes and macrophages are recognized as central players in the resolution and regulation of the inflammatory, proliferative, and remodeling phases of wound healing [29]. Unlike normal wound-infiltrating macrophages differentiating into classically-activated (inflammatory) M1 and alternatively-activated (reparative/regulatory) M2 macrophages, T2DM-affected macrophages strongly polarize into the inflammatory M1 phenotype [26]. Classically activated macrophages possess high phagocytic properties and are efficient in their production of pro-inflammatory cytokines—namely IL-1α, IL-1β, IL-6, TNF, and IL-12—contributing to and extending the inflammation phase, increasing neutrophilic infiltration, and prolonging low-grade inflammation which is characteristic of chronic DFUs [26,30]. Consequently, a lower absolute number of M2 macrophages and a higher M1:M2 macrophage ratio within the wound reduces secretory levels of growth factors PDGF, FGF, and VEGF, as well as anti-inflammatory cytokines including IL-10, TGF-α and TGF-β—all of which are responsible for the induction of the proliferative phase and effective regulation of inflammation, respectively [13,31]. Moreover, monocytes/macrophages act as antigen-presenting cells, linking the innate and adaptive immune responses [29]. Despite this, to date, the role of mutual interactions between macrophages and T cells in wound healing has yet to be fully elucidated.

It is well established that skin resident T cells play an essential role in the maintenance and regulation of local skin inflammation in the course of wound healing. In fact, Th17 cells were shown to promote neutrophilic infiltration, and high levels of IL-17A were shown to reduce wound repair [32]. On the other hand, regulatory T cells (Tregs) are considered essential regulators of inflammation and constitute a significant source of IL-10 [33]. Importantly, depletion of Tregs significantly reduces wound closure [34]. It is tempting to speculate that the systemic inflammation observed in diabetic patients limits the migration of Tregs and increases the infiltration of Th17 cells in the diabetic wound and thus represents one of the mechanisms of increased neutrophilic inflammation and a prolonged inflammatory phase. Notably, the healing process of diabetic wounds may be accelerated by topical retinoic acid, thereby inducing T cell plasticity and differentiation of Th17 cells towards Tregs [35]. This confirms the crucial role of T cells in the regulation of the inflammatory phase of diabetic wound healing.

Taken together, the prolonged inflammation phase observed in diabetic wounds that impairs wound closure and remodeling originates not only from high levels of localized pro-inflammatory mediators, but also from deficiencies in anti-inflammatory cytokines derived by regulatory cells, including M2 macrophages and Tregs. Despite this observation, the regulation of this process needs attention in future research.

## 4. Proliferation of Tissue Resident Cells Is Crucial for Wound Closure

Following the inflammatory phase of wound healing, the proliferative phase, characterized by formation of granulation tissue, begins (Figure 1). Granulation tissue consists of fibroblasts, immune cells, and newly formed blood capillaries which allow epithelial cell migration towards the apical wound surface in the process of re-epithelization [36]. As mentioned, fibroblasts, keratinocytes, mast cells, and M2 macrophages display potent regenerative activities, mainly through the secretion of cytokines (e.g., IL-10 and IL-35), growth factors (e.g., TGF-α, TGF-β, FGF and EGF), chemotactic factors for stem and progenitor cells (e.g., CXCL8 and SDF-1), and extracellular matrix reorganization through the activities of MMPs and their inhibitors (TIMPs) [13,23,29,30,31,32,37,38,39]. As discussed above, wounds with impaired healing kinetics and chronic wounds, including DFU, are known to significantly reduce skin-resident cell proliferation as well as stem and progenitor cell activation. Although this is partially the effect of the extended inflammatory phase, other compounding factors such as T2DM-mediated glycation of proteins, reduced angiogenic capability, and resultant oxidative stress contribute to the unnecessary extension of the proliferative phase, with the wounds failing to achieve closure in the most severe cases [26]. Detrimentally, the observation of dysfunctional and reduced numbers of circulating stem and progenitor cells, including endothelial progenitors, has been previously reported in T2DM patients [40,41]. This is in contrast to normally-healing wounds where neovascularization is the hallmark of the proliferation phase [4]. Contrastingly, the significantly reduced ability of endothelial progenitors to form new vessels accompanied by limited numbers of stem cells represents a significant contributor to disrupted re-epithelization in diabetic wounds [42,43]. Recently, stem cell-based therapies, including those based on mesenchymal stem cell (MSC) application, have become an attractive treatment strategy for impaired wounds, including DFUs. Novel strategies and treatment options for diabetic wounds will be discussed in the following paragraphs.

Wound closure requires reconstruction of the dermis before epithelial coverage by migratory basal keratinocytes can take place [38]. This stage requires the reconstruction of the three-dimensional collagen structure of the dermis upon which subsequent cell populations are located. Therefore, fibroblasts and myofibroblasts are considered central players in this process [44]. Their function is also supported by wound-resident macrophages, mast cells, and lymphocytes, in VEGF and TGF-β dependent mechanisms [29,30,31]. Importantly, this process is also closely associated with neovascularization of the wound bed, providing crucial nutrient and oxygen supplies to the healing site [45]. M2 macrophages additionally support wound angiogenesis by direct (macrophage-to-endothelial cell adhesion) and indirect (paracrine effect) mechanisms [46]. Interestingly, these activities are similar to those observed in tumor-associated macrophages, as recently discussed elsewhere [47,48,49]. Unfortunately, as mentioned before, in diabetic wounds, monocyte polarization towards M2 macrophages is inhibited, and pro-inflammatory polarization is promoted. Similarly, T2DM-impaired fibroblasts display a low activation level, decreased collagen deposition, and reduced paracrine signaling ability, including downregulation of TGF-β pathway activation [50].

A well-regulated proliferative phase is arguably the most crucial indicator of a successfully-healing wound, given the importance of both angiogenesis in tandem with epidermal coverage of the wound. Furthermore, when dysregulated, this process slows or even halts entirely, resulting in chronic ulcerative wounds. Although this mechanism remains to be fully elucidated due to the high number of participating entities, key cellular and molecular factors have been implicated in T2DM-induced or otherwise impaired wounds—namely fibroblasts, macrophages, key aforementioned growth factors, and unresolved/self-renewing inflammatory bodies from a persistent inflammatory phase.

## 5. Wound Remodeling

In the fourth phase of the healing process—wound remodeling—granulation tissue is strengthened by the accumulation of ECM proteins, which form scar tissue [44] (Figure 1). Moreover, the decrease of cellular and vascular components as well as an increase in the concentration collagens is observed with the principal aim of recovering normal skin function.

As with their strong participation in the proliferative phase, wound-resident fibroblasts, myofibroblasts, and M2 macrophages play an integral role in remodeling [38,44,51]. Collagens comprise 85% of the dermis and are consistently re-organized during wound healing to determine terminal scar fate after the remodeling phase [52]. At this stage of normal wound healing, collagen III undergoes degradation, with the subsequent deposition of type I collagen controlled by TGF-β and FGF signaling [53]. Similarly, secreted matrix metalloproteinases (MMPs) and their inhibitors (TIMPs) are able to guide the deposition and extraction of ECM components [54,55]. In this way, fibroblasts and macrophages can shape the final structure of a healing wound, with collagen fibers becoming thicker, denser, and intertwining—resulting in enhanced scar tissue strength in the normally-healing wound [56].

Notably, the ECM is dynamically subjected to constant changes throughout the remodeling phase, which results in the maturation of its structure. Its composition plays an essential role in skin repair via interactions of its protein structures, such as provisionally-deposited fibronectin and vitronectin, with different cell types [38]. Importantly, the delivery of ECM elements of decellularized skin structures during wound healing has been demonstrated to improve the wound healing process, and consequently become an attractive therapeutic approach by members of our and other groups (please see following chapters of this manuscript) [57,58,59,60,61].

In contrast to normally healing wounds, T2DM-affected wounds possess many structural and functional differences by comparison. Namely, abrogated angiogenesis resulting in a hypoxic wound environment and subsequent oxidative stress [26]. Recently, T2DM was shown to drive M1 macrophage polarization in the healing wounds of mice as a consequence of oxidative stress [26]. As previously mentioned, M1 macrophages promote inflammation and, their persistently increased numbers result in the differential expression of MMPs and TIMPs which are responsible for the reorganization of provisional ECM components in the late proliferative and remodeling phases of wound healing [38,62]. Therefore, guidance of tissue-infiltrating and resident macrophages towards a non-classical M2 phenotype, either by increased angiogenesis or elimination of oxidative stress, can result in a return to normative wound healing [26].

Similarly, fibroblasts in impaired wounds have their ECM deposition abilities significantly diminished. Using a 3D in-vitro culture, DFU-derived fibroblasts were observed to produce ECMs twofold thinner than normal [63]. Additionally, these thinner matrices were also shown to possess a greater composition of collagen type I and fibronectin content [63]. Additionally, topically-applied fibronectin has been previously shown to increase wound healing ability in DFUs, increasing angiogenesis while reducing inflammatory cytokine expression, apoptosis, and oxidative stress [64]. Taken together, these observations suggest that enhanced and/or corrective fibroblast activity can be potentiated by treatment with ECM components to compensate for the deficiency present in DFU fibroblasts.

## 6. Treatment Strategies for Diabetic Wounds and Ulcers

Our growing understanding of wound healing mechanisms has led to the development of a variety of potentially effective treatment strategies for hard healing wounds. Currently, well-established treatments for DFUs (standard care) include pressure off-loading from the wound site, debridement of necrotic tissue, pathogenic suppression, and topical wound dressings of varying types to minimize patient non-compliance and subsequent poor clinical outcome [65]. Frequently, these measures are used as control treatments in the evaluation of novel experimental therapies, although the material, treatment period, and other factors vary according to the type and severity of evaluated wounds [66,67].

Notably, experimental strategies (Table 1) include the (a) application of cell-based therapies—aimed at the systemic or local application of cells with regenerative potential (mainly stem and progenitor cells); (b) use of biologically-derived therapeutics; (c) application of physical methods such as hyperbaric pressure, electrical stimulation); (d) use of dermal and epidermal skins substitutes; and (e) combination of these strategies in addition to standard care [4]. In fact, skin substitutes and biological dressings are readily available and considered safe, promising options to treat large skin defects and hard-healing wounds. Therefore, in the following section, we will discuss the use of dermal scaffolds and dressings in the context of diabetic wound healing.

## 7. The Use of Skin Substitutes in Diabetic Wound Healing

Skin substitutes can be divided into subcategories based upon their composition, derivative source, and unique additives, if any exist (Table 2). Among implantable scaffolds, these include those with a dermal and/or epidermal component. Further distinguishment can be observed based on whether the biomaterials used are derived from a biologic source, fully synthetic, or a mixture of both. Further disambiguation can occur as to whether the materials are derived from the host of the transplant (autogenic), another human donor (allogenic), or derivative of another animal species (xenogenic). Lastly, scaffolds may be classified by whether they are completely acellular or not, with non-autologous cellular matrices theoretically possessing the risk of an adverse reaction as a consequence of host rejection. Notably, however, many successful skin substitutes are composed of a variety of individually-sourced materials, obfuscating the full contributory mechanism of individual components [76,77]. This is especially true given the complexity of chronic wound environments and the clinical variability within an individual at the local and systemic level. Hereafter we will focus on scaffolds which possess a dermal element—in particular fully acellular dermal matrices—due to dermal element (1) prevalence in DFU treatment, (2) ECM-related therapeutic mechanistic effects induced, (3) safety in regard to tissue rejection, 4) history of beneficial clinical outcomes, and (5) their abundance in recent clinical trials (Table 1).

## 8. Dermal Scaffolds

Dermal scaffolds are dermal tissue-derived or dermis-like matrices that retain the ability to integrate into host ECM or are cleaved, thereby supporting re-epithelization and maturation of the healing wound [78]. Given the extensive role that the ECM plays in wound healing, the examination of wound substitutes that mimic native dermis has been implicated as an effective ameliorative therapy, often in conjunction with supplementary cellular or molecular components [76,79,80]. Consequently, recent decades have seen innovation in 3D cell cultures and other substitutes which model human skin in vitro, allowing for the evaluation of skin substitutes more readily than with animal models alone [81]. Despite this, some skin substitutes (including dermal scaffolds) do not possess the ability to fully integrate with host fibroblast-derived ECM components, often leading to future complications in their extraction or inability to undergo desirable ECM/collagen deposition during the remodeling phase of wound healing. However, recent evidence has demonstrated the association of dermal scaffolds with beneficial therapeutic outcomes—particularly in impaired wound resolution [82,83].

Tissue engineering has become a valuable tool in the creation of scaffolds that can integrate with a recipient’s tissue, due in large part to recent innovations in engineering technology and the success of traditional biologically-sourced scaffolds. Therefore, the creation of biomimetic engineered artificial, synthetic, or natural substitutes for bone, skin, and/or blood vessels has shown a marked interest. In fact, ideal scaffolds and tissue substitutes including skin matrices, be they bio-engineered or natural, should be characterized as: low- or non-immunogenic, bio-compatible, regenerative, protective, non-pathogenic, and durable (Table 3). In fact, acellular dermal matrices possess many of these characteristics and are considered useful dressings in skin wounds, including hard healing or non-healing wounds such as DFUs. Their therapeutic properties originate from and depend on their source, method of preparation, and further modification. Therefore, in the following part of this review, we will summarize current knowledge on the use of acellular dermal matrices and cell-covered dermal matrices in wound healing.

## 9. Acellular Dermal Matrices

Acellular dermal matrices are perhaps the most biomimetic scaffolds, as they can retain the primary functional structure of normal dermal tissue. This is particularly important because, as mentioned above, intact ECM components strongly affect the healing potential of a wound and its subsequent reorganization during remodeling [52]. However, the method of decellularization and tissue source must be addressed, given that this process differentially affects the wound microenvironment [84] by way of retaining functional matrix proteins and components, as well as its physical characteristics—as in the case of collagen fiber cross-linkage [85,86].

Given that the most abundant dermal collagen is type I by a wide margin, followed by type III, acellular dermal matrices (ADMs) are generally similar in composition. As a consequence of their abundance and early research demonstrating the chemotactic attraction of human fibroblasts to collagens I, II, and III [87], they receive a great deal of attention. Notably, monocyte adhesion to collagen types I and III have a demonstrable effect on the secretion of the wound and ECM-affecting products including growth factors [88,89,90], cytokines [91], and enzymes [89] which play a crucial role in normative wound healing [31,92,93,94]. Similarly, 3D environments dense with collagen I fibers were recently shown to induce immunosuppressive effects in M2 macrophages [95], suggesting a beneficial therapeutic effect in DFU-resident macrophages. This is further supported by the observation that the overabundance of type III collagen, seen in hypertrophic wound scars is by itself insufficient to induce immunomodulatory effects sufficient to resume normative wound healing, as has been previously observed with collagen type I deposition [96].

Although the full contributory role of ADMs in DFUs has not yet been elucidated, the mechanistic effects of specific proprietary ADMs has been witnessed. In one study, application of a xenogenic ADM was able to return the M1:M2 macrophage polarization ratio normally seen in DFUs to that of normally-healing wounds [97]. Furthermore, ADMs have also been witnessed to induce increased levels of microvascular blood flow within DFUs [98]. These promising results firmly establish ADMs as favorable candidates for further research in the context of chronic wounds, especially given the crucial importance of macrophage ratio and angiogenesis therein.

Due to their ubiquity and history of effective clinical outcomes, ADMs are frequently utilized in a variety of pathologies besides DFU, including rare skin conditions, such as epidermolysis bullosa, plastic surgery, and burn treatment, among others [59,60,99,100]. Recent studies and meta analyses have shown the effectiveness of ADMs in regard to their ability to influence the immune response by differentially modulating key growth factors and cytokines, resulting in enhanced wound closure and faster resolution [57,61,101].

By highly mimicking normal dermal tissue and subsequently eliminating potential immunogenic antigens on the surface of donor cells, it is hoped that these scaffolds will induce a return to normative wound healing in the recipient. This is further buttressed by observable differences in cellular vs. non-cellular human dermal matrices—underpinning the importance of cell-associated immunogenic component removal as a means to minimize ADM rejection and associated complications [102,103].

Likely obfuscated by the inflammatory nature of DFUs, ADM-mediated immunogenic responses go largely unnoticed. However, this phenomenon can occasionally be witnessed in sterile, normally-healing wound environments such as breast reconstruction in the form of red breast syndrome (RBS). Although the etiology of RBS is speculative, it is limited in nature—resolving without treatment and theorized to clear as a consequence of ADM neovascularization [104]. RBS and other ADM-related adverse effects, although very rare, are likely related to the presence of endotoxin or wound contamination with microbes [105]. Despite the likelihood that ADM-treated DFUs face a similar sterile inflammatory response, ADMs have been witnessed to be exceedingly safe, with high healing rates vs. standard care and a lack of immunogenic, toxic, or carcinogenic complications [106].

Although the key elements regulating skin substitute-mediated wound healing mechanisms remain to be fully elucidated, recent evidence has shed light on essential modulations in the wound microenvironment, which subsequently lead to beneficial therapeutic outcomes. It seems that the induction of differing ADM physical characteristics and mechanistic effects within the wound micro-environment are based not only upon the source, but crucially on the method of preparation that proprietary ADMs undergo—namely in relation to the decellularization and sterilization processes.

Notably, irradiation for sterilization purposes may fundamentally damage or change the structural components of skin substitutes depending on dose [85,86]. This process induces structural changes and can result in the damage and extraction of ECM components which may directly affect the healing process supporting the epithelization process and remodeling or inducing inflammation. In fact, it appears that modulation of scaffold degradability, among other physical characteristics, can be cultivated via physical and chemical modification in order to facilitate guided responses, including the infiltration, adhesion, and proliferation of regenerative cells [107]. On the other hand, when adversely affecting the therapeutic potential of biological dressing, the sterilization process may be bypassed by appropriate aseptic production, assuming that proper precaution against and screening for endotoxins is undertaken [106].

## 10. Cell-Supplemented Dermal Matrices

Having established the high customizability and effectiveness of keystone elements of ADMs, on their own they hold a great deal of potential. Even so, skin substitutes’ therapeutic potential may be improved by the supplemental utilization of stem and progenitor cells with well-characterized activities supporting the healing process, as in the case of mesenchymal stem cells (MSCs) and fibroblasts [103,108,109]. Interestingly MSCs co-cultured with skin substitutes in vitro were shown to release trophic factors important for tissue regeneration [110] and to improve the healing of diabetic wounds when used in tandem skin substitutes, including ADMs [76,79,110,111,112]. This strong regenerative effect is associated primarily with MSC ability to improve neovascularization of the wound via paracrine activity in addition to their well-characterized anti-inflammatory effects [113]. Furthermore, the lack of notable differences in clinical outcomes when comparing autologous and allogeneic MSCs, suggests their low immunogenicity [114], leading to speculation as to entirely allogenic therapeutic possibilities without the risk of tissue rejection. In fact, a recent systematic review of adipose-derived stem cells used in conjunction with ADMs found them to be both safe and effective [115]. These promising results indicate the efficacy and safety of MSC-mediated therapy for DFUs, with the number of active clinical trials including MSCs in DFUs (Table 1).

Presently, acellular dermal matrices hold a great deal of promise for the treatment of hard-healing wounds, including diabetic wounds [57,99,100,101]. Their continued usage and examination in regard to source tissue, structural composition, and preparation are of supreme importance, with these factors playing key roles in the degradation of specific collagen fibers and their ability to integrate and provide the most beneficial immunomodulatory effects. Further, given the beneficial effects associated with additive cellular and molecular components such as growth factors, proteases, and easily-attainable MSCs, combinations of these additives with ADMs have been evidenced as safe and effective treatments leading to favorable therapeutic outcomes in DFUs, especially when compared against current standards of care (Table 1).

## 11. Conclusions

Diabetic wounds remain a significant clinical problem. The understanding of complex mechanisms of stem and progenitor cell dysfunctions and the dysregulation of systemic and local immune responses will significantly contribute to the efficacy of currently used therapies. However, the use of biological dressings, such as skin substitutes, additionally supported by stem cells or stem cell derived-fragments may represent a readily accessible and advantageous option for treating diabetic wounds. Notably, more studies focusing on specific biomaterials and their contributory influence to specific elements of the wound microenvironment are preferred. Effective guidance of skin substitute characteristics and the mechanistic contribution therein will help to develop innovative and effective protocols to treat chronic wounds in diabetic individuals well into the future.

## Figures and Tables

**Table 1:** Clinical trials involving biological materials including or with potential secondary application with skin substitutes and acellular dermal matrices in diabetic foot ulcers and impaired wounds.

	Name of Clinical TRIAL	Last Update	Clinical Trial ID	Status	Conclusions	Publications (PMID)
Acellular Dermal Matrices	Effect of Meso Wound Matrix in the Treatment of DFUs	22 October 2020	NCT04182451	Active, not recruiting	No results available	
	Comparative Effectiveness of Two Acellular Matrices (Dermacell vs. Integra) for Management of Deep Diabetic Foot Ulcers	2 September 2020	NCT03476876	Recruiting	No results available	
	DermACELL AWM^®^ in Chronic Wagner Grade 3/4 Diabetic Foot Ulcers	6 September 2019	NCT03044132	Completed	DermACELL healed complex DFUs with exposed bone. Results suggest wound closure if given time.	[68] 31361269
	DermACELL in Subjects With Chronic Wounds of the Lower Extremities	14 March 2018	NCT01970163	Completed	DermACELL increases in healing rates in DFUs compared with conventional care options	[69] 26933467
					DermACELL-treated subjects had higher wound closure than those treated with ADM Graftjacket.	[70] 28544150
	OASIS Wound Matrix (Oasis) Mechanism of Action	9 June 2011	NCT00570141	Completed	7 of 13 wounds closed fully after 12 weeks.	
	Acellular Porcine Dermal Matrix Wound Dressing in the Management of Diabetic Foot Ulcers	7 June 2011	NCT01353495	Completed	Submitted; Pending	
Skin Substitutes	AMNIOEXCEL^®^ Plus vs. A Marketed Comparator vs. SOC in the Management of Diabetic Foot Ulcers	20 July 2020	NCT03547635	Completed	13,000 treatments: All matrices roughly equivalent in closure over 12 weeks. SIS & UBM healed more quickly and cost less.	[71] 27681811
					Half of patients treated achieved wound closure vs. none with SOC	[72] 26978860
	Multi Center Site, Controlled Trial Comparing a Bioengineered Skin Substitute to a Human Skin Allograft	26 June 2019	NCT01676272	Completed	No results available	
	Clinical Outcomes After Treatment With RestrataTM Wound Matrix in Diabetic Foot Ulcers (DFU)	13 August 2018	NCT03312595	Completed	No results available	
	Pivotal Trial of Dermagraft(R) to Treat Diabetic Foot Ulcers	21 May 2018	NCT01181453	Completed	Benefit for chronic DFUs >6 weeks duration	[73] 12766097
	Dermagraft(R) for the Treatment of Patients With Diabetic Foot Ulcers	21 May 2018	NCT01181440	Completed	Dermagraft-treated patients have better healing than SOC.	[73] 12766097
Growth Factors	BB-101 (Recombinant Human for Treatment of Diabetic Lower Leg and Foot Ulcers	14 September 2020	NCT03888053	Recruiting	No results available	
	A Randomized Trial on Platelet Rich Plasma Versus Saline Dressing of Diabetic Foot Ulcers	16 September 2019	NCT04090008	Completed	No results available	
	Efficacy and Safety of Heberprot-P^®^ in Patients With Advanced Diabetic Foot Ulcer in Dasman Diabetes Institute.	4 August 2017	NCT03239457	Completed	No results available	
	A Phase 3 Clinical Trial to Assess the Effectiveness of BioChaperone PDGF-BB In the Treatment of Chronic Diabetic Foot Ulcer	29 June 2017	NCT02236793	Completed	No results available	
	A Study Evaluating Topical Recombinant Human Vascular Endothelial Growth Factor (Telbermin) for Induction of Healing of Chronic, Diabetic Foot Ulcers	11 May 2017	NCT00069446	Completed	No results available	
	Comparative Study of 3 Dose Regimens of BioChaperone to Becaplermin Gel for the Treatment of Diabetic Foot Ulcer	15 December 2014	NCT01098357	Completed	No results available	
	Efficacy and Safety of rhEGF in Diabetic Foot Ulcer Patients With Uncontrolled Diabetic Mellitus	4 August 2014	NCT01629199	Completed	No results available	
	Evaluation of the Safety Follow-up of Becaplermin or Placebo Gel Following Treatment of Chronic, Full Thickness Diabetic Ulcers	8 June 2011	NCT00740922	Completed	No results available	
	Gene Therapy to Improve Wound Healing in Patients With Diabetes	20 November 2007	NCT00065663	Completed	No results available	
Misc.	Utilization of Platelet Gel for Treatment of Diabetic Foot Ulcers	4 December 2015	NCT02134132	Completed	No results available	
	Evaluation of the Effect of Vivostat Platelet Rich Fibrin (PRF) in the Treatment of Diabetic Foot Ulcers	12 October 2011	NCT00770939	Completed	No results available	
MMPs	Matrix Metalloproteinase-1/Tissue Inhibitor of Metalloproteinase-1 (MMP-1/TIMP-1) Ratio and Diabetic Foot Ulcers (DIAB-MMP2)	18 December 2013	NCT00935051	Completed	No results available	
Mixed	Phase 2b Study of GAM501 in the Treatment of Diabetic Ulcers of the Lower Extremities (MATRIX)	10 February 2010	NCT00493051	Completed		[74] 17199833
Stem Cells	Phase 1, Open-Label Safety Study of Umbilical Cord Lining Mesenchymal Stem Cells (Corlicyte^®^) to Heal Chronic Diabetic Foot Ulcers	6 August 2020	NCT04104451	Recruiting	No results available	
	Clinical Study of Adipose-derived Stem Cells in the Treatment of Diabetic Foot	16 April 2019	NCT03916211	Not yet recruiting	No results available	
	Comparison of Autologous MSCs and Mononuclear Cells on Diabetic Critical Limb Ischemia and Foot Ulcer	1 December 2010	NCT00955669	Completed	BMMSCs led to increased blood flow and wound healing compared to BMMNCs	[75] 30917698
Endothelial Progenitor Cells	Cryopreserved Human Umbilical Cord (TTAX01) for Late Stage, Complex Non-healing Diabetic Foot Ulcers (AMBULATE DFU II)	24 November 2020	NCT04450693	Recruiting	No results available	
	Cryopreserved Human Umbilical Cord (TTAX01) for Late Stage, Complex Non-healing Diabetic Foot Ulcers (AMBULATE DFU)	5 November 2020	NCT04176120	Recruiting	No results available	
Antibodies	The Safety, Tolerability, Pharmacokinetics, and Pharmacodynamics of UTTR1147A in Participants With Neuropathic Non-Healing Diabetic Foot Ulcers	21 November 2018	NCT02833389	Completed	No results available	

* Table data taken from clinicaltrials.gov, updated on 17 January 2021. Key search words included: “Diabetic Foot Ulcer” and “Impaired Wound,” as well as associated subcategories.

**Table 2:** Categories of Skin Substitutes.

Composition	Material	Additives
Dermal	Autogenic	None—Fully Acellular
Epidermal	Allogenic	Acellular with Remnants
Full Skin	Xenogenic	Cellular (MSCs)
	Synthetic	Molecular (MMPs/TIMPs/Growth Factors/Cytokines)
	Mixed	

**Table 3:** Ideal properties of skin substitutes.

Property	Elaboration
Non-immunogenic	Components do not induce tissue rejection.
Bio-compatible	Infiltrating cells can effectively adhere to scaffold material. Integrates readily into existing ECM; cells can deposit/extract ECM
Regenerative	Does not inhibit or promotes angiogenic function Minimizes sub-optimal granulation of tissue & scarification Beneficially modulates regenerative cells such as macrophages & fibroblasts Facilitates rapid epithelial cell coverage
Protective	Provides coverage for underlying structures Minimizes disruptive “floating” in the wound bed Retains & maintains a moist environment, reducing oxidative stress
Non-pathogenic	The low number of applications to minimize infection risk Sterile preparation method & donor source do not confer disease
Durable	The substitute does not degrade before regenerative action. Complicating conditions (infection, T2DM-induced glycosylation, etc.) do not compromise scaffold flexibility & function
